# Coexistence of predators in time: Effects of season and prey availability on species activity within a Mediterranean carnivore guild

**DOI:** 10.1002/ece3.6778

**Published:** 2020-09-12

**Authors:** Marc Vilella, Mariona Ferrandiz‐Rovira, Ferran Sayol

**Affiliations:** ^1^ Delegació d’Osona (GNO‐ICHN) Institució Catalana d’Història Natural Vic Spain; ^2^ CREAF Cerdanyola del Vallès Catalonia Spain; ^3^ Universitat Autònoma de Barcelona Cerdanyola del Vallès Catalonia Spain; ^4^ Centre for Biodiversity and Environment Research Department of Genetics, Evolution and Environment University College London London UK; ^5^ Department of Biological and Environmental Sciences University of Gothenburg Gothenburg Sweden; ^6^ Gothenburg Global Biodiversity Centre Gothenburg Sweden

**Keywords:** activity overlap, activity pattern, camera trapping, mesocarnivore, relative activity index, small mammals, temporal niche

## Abstract

1. The degree of coexistence among predators can determine the structure of ecological communities. Niche partitioning is a common strategy applied by species to enhance their coexistence. Diet, habitat, or time use can be responsible for segregation among carnivore species, the latter factor being the least studied in Mediterranean ecosystems. Terrestrial medium‐sized carnivores (i.e., mesocarnivores) carry out important functions in ecosystems, and identifying their interactions is essential for their conservation.
2. In this study, we explore the activity of a terrestrial mesocarnivore guild in order to determine seasonal differences in daily activity patterns of competitors and prey. We also investigate how the abundance of a common mesocarnivore prey in the region, small mammals, influences the activity of predators.
3. During a year, camera trap devices (*n* = 18) were installed in Montseny Natural Park (Catalan Pre‐Coastal Range, North‐East Iberian Peninsula), a region that hosts five mesocarnivore species. Camera trapping detections were used to estimate their daily activity patterns and corresponding overlaps. We also surveyed small mammal plots (*n* = 5) in order to calculate prey abundance and test its effect on the relative activity of each carnivore species.
4. Despite all target mesocarnivores are mainly nocturnal, the activity overlap among them varies according to species particularities and season. Red fox (*Vulpes vulpes*) appears as a generalist species in terms of time use, whereas stone marten (*Martes foina*) and genet (*Genetta genetta*) show the most similar activity patterns and both of them seem to be positively influenced by small mammal abundance. Overall, the diversity found in the way mesocarnivore species use time could facilitate their coexistence.
5. Despite activity pattern similarities among carnivore species should not be directly translated to negative interactions, they can have a strong influence in habitat and resource‐limited ecosystems. Therefore, activity overlaps should be taken into account when discussing wildlife management actions.

## Graphical abstract

Here, we investigate how temporal niche dynamics of five Mediterranean carnivores can affect coexistence among intraguild species. We show differences on their time use that can facilitate co‐occurence and the influence of season and prey availability on it. Therefore, accounting for activity differences might help to better understand the diversity of intraguild species observed in an ecosystem.

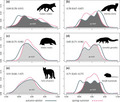

## 1 INTRODUCTION

Mammal carnivores play an important role in terrestrial landscapes, as their top‐down effects can regulate prey populations with consequences spreading across the entire food web (Roemer, Gompper, & Van Valkenburgh, 2009; Schmitz, Hambäck, & Beckerman, 2000). Consequently, they are commonly identified as umbrella species that promote the protection of entire ecosystems (Roberge & Angelstam, 2004), thus being targeted for conservation efforts (Karanth & Chellam, 2009). In addition, some studies suggest the protection of entire carnivore guilds in order to preserve their ecosystem function (Dalerum, 2013; Dalerum, Cameron, Kunkel, & Somers, 2009). However, protecting multiple carnivore species can be challenging if species exhibit strong competitive interactions that compromise their coexistence (Fedriani, Fuller, Sauvajot, & York, 2000; Linnell & Strand, 2000).

Interspecific competition for resources affects all trophic levels (Chesson & Kuang, 2008; Menge & Sutherland, 1976), but it is especially intense among predators due to their large requirements (Fedriani et al., 2000). Terrestrial carnivores have usually adapted their behavior in order to reduce negative interactions among them (Linnell & Strand, 2000), a process known as niche partitioning. Several niche axes that could play a role in carnivore coexistence have been analyzed in a variety of ecosystems. Among them, land cover selection and diet preferences are some of the most studied factors (Barrientos & Virgós, 2006; Carvalho & Gomes, 2004; Curveira‐Santos, Pedroso, Barros, & Santos‐Reis, 2019; Kelly & Holub, 2008; Linkie, Dinata, Nugroho, & Haidir, 2007; Rosalino, Macdonald, & Santos‐Reis, 2004; Ruiz‐Olmo & López‐Martín, 2001; Torre, Arrizabalaga, & Ribas, 2009; Virgós, Romero, & Mangas, 2001). However, despite similar diet or habitat requirements, time partitioning could favor the coexistence of species that occupy the same guild. Due to the popularization of camera trapping as a technique to study wildlife, the number of studies focusing on the temporal niche of sympatric carnivores has recently increased (Azevedo, Lemos, Freitas‐Junior, Rocha, & Azevedo, 2018; Bu et al., 2016; Karanth et al., 2017; Massara, Paschoal, Bailey, Doherty, & Chiarello, 2018; Mukherjee et al., 2019; see also Meredith & Ridout, 2018 and references therein). Some of them show that temporal avoidance can reduce competition and thus facilitate species coexistence (Hearn et al., 2018; Herrera et al., 2018; Karanth et al., 2017; Lucherini et al., 2009; Massara et al., 2018; Mukherjee et al., 2019), especially in cases where species segregate between diurnal, crepuscular, and nocturnal domains (Marinho, Fonseca, Sarmento, Fonseca, & Venticinque, 2020; de Satgé, Teichman, & Cristescu, 2017; Zhao et al., 2020). These studies highlight the need to account for activity overlaps in order to gain knowledge on the interactions of competing carnivores. However, as most of this work on time use has been applied to tropical landscapes, there is still a lack of information for many temperate species, especially within the Mediterranean area (but see Barrull et al., 2013; Curveira‐Santos, Marques, Björklund, & Santos‐Reis, 2017; Monterroso, Alves, & Ferreras, 2014; Torretta, Serafini, Puopolo, & Schenone, 2016).

One way to evaluate the temporal niche of a population is the daily activity pattern (i.e., the relative activity of the population at each moment of the day). Daily activity patterns of many large, medium, and even small mammal species (Torre, 2004) can be estimated by means of camera trapping, as it enables obtaining precise dates and times at which individuals are active. Subsequently, temporal or activity overlaps—defined as the similarity between two activity patterns—can be also calculated. These similarities are usually described by using three variables: activity levels (i.e., the number of hours in a day that a population is active) (Rowcliffe, Kays, Kranstauber, Carbone, & Jansen, 2014), activity period (i.e., the exact hours at which the population is active), and activity peaks (i.e., specific hour of maximum activity). Camera trapping also enables to obtain a Relative Activity Index (RAI), an indirect measure of species activity estimated by the number of independent detections within a time interval (Bu et al., 2016). Species RAI complements the information on daily patterns as it explores activity at a larger temporal scale. Finally, some other advantages of using camera trapping are a reduction in human sampling error and an increase in monitoring scales (Ahumada et al., 2011; Barea‐Azcón, Virgós, Ballesteros‐Duperón, Moleón, & Chirosa, 2007; Burton et al., 2015; Peris, Tena, & Villena, 2011).

The way carnivores use time can vary according to a number of factors, such as meteorology, the presence of large apex predators, human frequentation, or even ancient selective pressures that are not currently present (Kronfeld‐Schor & Dayan, 2003; Parsons et al., 2016; Rosalino, Macdonald, & Santos‐Reis, 2005; Suraci, Clinchy, Dill, Roberts, & Zanette, 2016). However, apart from daylight variations across the year and other aspects derived from seasons (Zhao et al., 2020), species diet is one of the factors that could have the strongest influence on carnivore activity (Penido et al., 2017). For instance, species with generalist diets can keep a relatively high daily activity overlap among them because there is less risk of developing exploitation competition (Bu et al., 2016). Moreover, carnivore activity might be influenced by prey fluctuations, as the most specialized predators tend to adapt their patterns according to them (Azevedo et al., 2018; Brown, Kotler, & Bouskila, 2001; Foster et al., 2013; O’Connell et al., 2006). Finally, this overlap between predator and prey daily activity patterns can vary across time and space according to ecosystem dynamics (Barrull et al., 2013; Brown et al., 2001; Bu et al., 2016). For instance, a more intense relationship between predator and prey is expected when communities are regulated in a bottom‐up manner, a typical situation that occurs in locations with resource‐limited conditions during some periods of the year, such as the Mediterranean region (Meserve, Kelt, Milstead, & Gutiérrez, 2003).

The main objective of this study was to investigate the temporal niche partitioning process in a terrestrial mesocarnivore guild that inhabits a Mediterranean ecosystem. To fulfill this aim, we deployed 18 camera trap stations in Montseny Natural Park (Catalan Pre‐Coastal Range, North‐East Iberian Peninsula) during 1 year and estimated two different activity variables of mesocarnivore species: one at a daily level (activity pattern) and the other at a yearly scale (Relative Activity Index—RAI). From the former, we analyzed the activity overlap within the mesocarnivore guild and between predators and a common mesocarnivore prey in the region: small mammals. In addition, we collected data on small mammal abundance in five different plots to estimate how it might influence the relative activity of predators. The results of this work highlight the importance of identifying the way carnivores use time and the variables that influence it in order to understand potential intraguild coexistence issues and define appropriate conservation measures to face them.

## 2 MATERIALS AND METHODS

### 2.1 Study area and target species

**Figure 1:**
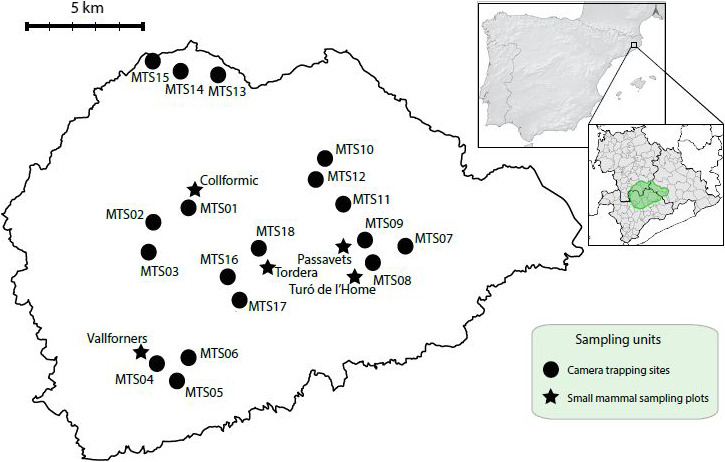
Montseny Natural Park position within the Iberian Peninsula, indicated as a green polygon at a regional scale. Locations of camera trapping sites, represented by the mean coordinates obtained from the different device positions within each of them, and small mammal sampling plots are symbolized according to the legend

Data were collected in Montseny Natural Park (Catalan Pre‐Coastal Range, North‐East Iberian Peninsula) during a whole year, from July 2018 to July 2019. Montseny is the largest and highest massif of the Catalan Pre‐Coastal Range, halfway between the Mediterranean Sea and the Pyrenees (Figure 1). Montseny stands out by its large altitude range, from 150 to more than 1,700 m. Consequently, it offers an extended gradient of environmental conditions and habitats within a continuous protected area of 55,090 ha. Despite the fact that 51,760 people live in this area (~100 people km^−2^), traditional human activities such as agriculture or ranching are not currently much spread. Instead, forest areas cover almost 80% of the surface, being sclerophyll tree species the most abundant—*Quercus ilex* and *Q. suber* (Torre et al., 2009).

As in many other areas within the Eastern part of the Iberian Peninsula, large predator species—Iberian lynx (*Lynx pardinus*), Eurasian lynx (*Lynx lynx*), brown bear (*Ursus arctos*) and gray wolf (*Canis lupus*)—are absent from the massif at least since the last century (Ruiz‐Olmo & Aguilar, 1995). Consequently, the apex of the regional trophic chain is occupied by five terrestrial mesocarnivore species, all of them included in the study: red fox (*Vulpes vulpes*), stone marten (*Martes foina*), European badger (*Meles meles*), common genet (*Genetta genetta*), and European wildcat (*Felis silvestris*). Small mammals are one of the most common prey consumed by these predators in Mediterranean habitats (Carvalho & Gomes, 2004). However, regarding small mammal percentage in relation to overall prey biomass in locations that are similar to the study area, the five mesocarnivores could be divided in three groups: species with occasional consumption of small mammals (badger—<10% according to Rosalino, Loureiro, Macdonald, & Santon‐Reis, 2005), species with regular consumption (red fox and stone marten—around 30% according to Padial, Ávila, & Gil‐Sánchez, 2002), and species with specialized consumption (wildcat and genet—from 80% to 90% according to Ruiz‐Olmo & Aguilar, 1995 and Torre, Ballesteros, & Degollada, 2003, respectively). Other resources exploited by these species are birds (especially the stone marten), invertebrates (typically the badger), or carrion (mainly the red fox). Finally, fruits are often consumed by red fox, stone marten and badger (Carvalho & Gomes, 2004; Rosalino, Loureiro, et al., 2005). In relation to the habitat, all five mesocarnivores are typical of Mediterranean ecosystems, especially the forested ones (Barrull et al., 2013; Ferreras, Díaz‐Ruiz, Célio, & Monterroso, 2016; Rosalino et al., 2004; Santos‐Reis et al., 2004). Besides, no important avoidance behavior has been reported among these five species at a broad scale, thus coexisting in many large areas (Barrull et al., 2013; Ferreras et al., 2016). Finally, home range size is also similar among them: stone marten, genet, and wildcat mean home ranges are estimated to measure between 2 and 3 km^2^ (Monterroso, Brito, Ferreras, & Alves, 2009; Santos‐Reis et al., 2004); while badger and red fox tend to have slightly larger territories: mean size between 4 and 5 km^2^ (Ferreras et al., 2016; Rosalino et al., 2004).

### 2.2 Camera trapping

Camera trap devices were set in 18 different sampling sites (these units will be referred to as “camera trapping sites” hereafter), trying to maximize the geographical and habitat range of Montseny massif covered. As a result, we sampled a considerable part (533–1,465 m) of the altitude gradient of the protected area. Cameras were always installed in evident fauna trails located at suitable forested habitats in order to favor mesocarnivore detectability (Curveira‐Santos et al., 2019; Karanth et al., 2017; Linkie et al., 2007; Mukherjee et al., 2019; Zielinski & Kucera, 1995). Therefore, note that we were mainly recording activity patterns derived from animal movements across forested sites, as opposed to activity in other contexts, such as hunting in open fields. To facilitate camera revisions, site distribution drew six groups (Figure 1). However, the 18 camera trapping sites were always located at a minimum distance of 800–1,200 m from each other, a sufficient interval to theoretically assume independence among them (Kelly & Holub, 2008; Moruzzi, Fuller, DeGraaf, Brooks, & Li, 2002; O’Connell et al., 2006).

Devices belonged to two different brands: Cuddeback (*C1* and *C2,* models that only differ in the flash type) and Browning (*Strike Force HD Pro*). Cameras were set to operate 24 hr a day, to take three photo‐bursts every time the sensor was triggered and to apply the minimum trigger delay possible. Camera time was set according to Coordinated Universal Time (UTC), as the time standard approaches true or apparent solar time the most in the region. No lures or baits were used as the aim was to capture species natural behavior: Some authors reported that attractors can have different effects depending on individuals and species (Barea‐Azcón et al., 2007; Rovero & Zimmermann, 2016; Torre et al., 2009). Accounting for local landscape irregularities (slope, edges, etc.), devices were placed at around 30 cm (exceptionally up to 100 cm) above the ground in order to focus on the images at a height of 20–50 cm, according to the size of the target species. Finally, they were secured and vegetation around them was cleared to avoid false triggering when necessary.

Most of the cameras operated continuously for nearly a year (328.28 ± 50.78; mean nights camera^−1^ ± SD), resulting in a total of 5,909 trap nights, expected to be enough to get a considerable number of target species detections (Burton et al., 2015). Devices were revised once every 30–40 days to obtain the images and check batteries. In order to minimize the bias that might produce the camera position itself—for instance, if by chance a camera was placed near a badger sett, badger activity would be overestimated—devices were moved within each site to 50–150 m from the initial position every 12–20 weeks, assuring that these changes did not coincide with season solstices or equinoxes.

**Figure 2:**
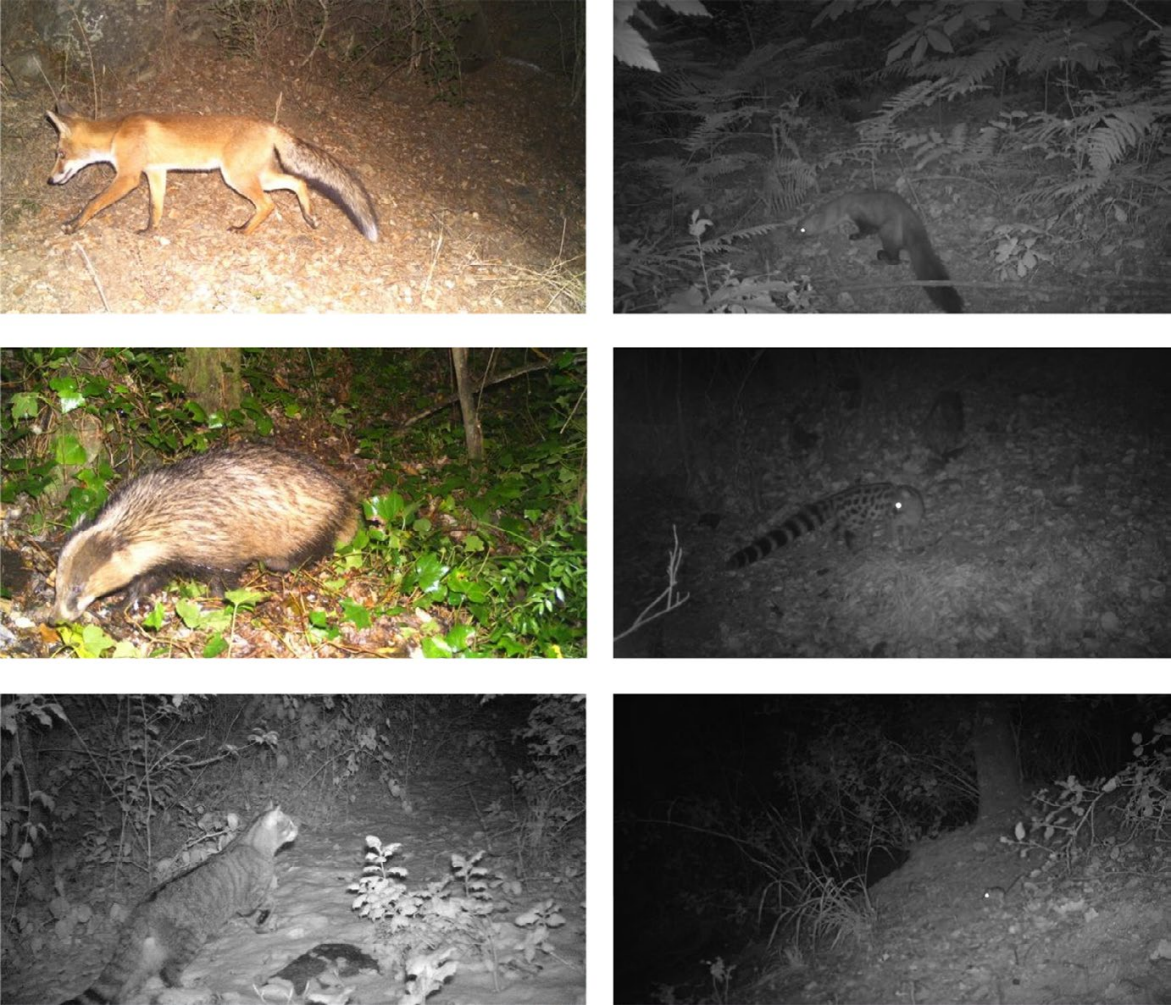
Example of some camera trapping pictures obtained during the study. From top left to bottom right: red fox *Vulpes vulpes*, stone marten *Martes foina*, European badger *Meles meles*, common genet *Genetta genetta*, European wildcat *Felis silvestris*, and wood mouse *Apodemus sylvaticus*

Mesocarnivore pictures taken by camera traps were identified at species level but not for small mammal ones, in which species was not evident in most cases (Figure 2). Different individuals of the same species were not identified due to the absence of individual fur marks in four of the five taxa. The rare cases in which more than one individual of the same species was present in a picture were considered as single detections in order to facilitate the analysis. Picture metadata was extracted with *ExifTool* application and used to build a database including camera trapping site, species, and date and time of each picture by using the R package *camtrapR* (Niedballa, Sollmann, Courtiol, & Wilting, 2016). Two pictures of the same species at the same camera were only considered as independent detections (contacts) if they were separated by a minimum time interval of 30 min. This interval—together with the 1‐hr interval—is commonly used in camera trapping studies and enabled us to maximize the number of theoretically independent contacts, allowing a more precise estimation of daily activity patterns (Azevedo et al., 2018; Bu et al., 2016; Jiménez et al., 2017; Kelly & Holub, 2008).

### 2.3 Small mammal sampling

Five small mammal sampling plots were set within the area (see Figure 1), and data were collected in two campaigns lasting three nights each, one during November–December 2018 and the other during June–July 2019. These plots are part of a larger network that aim to monitor small mammal biodiversity (SEMICE Project) in the Mediterranean region (Torre, Arrizabalaga, Freixas, Pertierra, & Raspall, 2011)—three of them have been running for some years, whereas two additional ones were set for the purpose of this study, following the guidelines of the project (Torre et al., 2011). Each plot consisted of 36 live traps (18 Sherman and 18 Longworth model units), which were distributed either in a 6 × 6 or in a 9 × 4 grid depending on landscape features and always with a distance of ~15 m between traps, covering a total area of 0.56 ha. All traps were filled with a mixture of flour and tuna in oil as bait and a piece of apple (ca. 10 g) to ensure enough individual hydration during the capture period, as the piece is never totally consumed. Besides, a handful of hydrophobic cotton was added so that the captured individuals could make a nest and feel safe. Traps were checked at dawn so that small mammals were inside the traps the minimum time possible. All individuals were identified at species level, marked with an ear tag and released. Capture and handle small mammals authorization was issued by Generalitat de Catalunya (Departament de Territori i Sostenibilitat) with identification code SF/0554/2019. All the handling and sampling adhered to the ASAB/ABS Guidelines for the Use of Animals in Research. Assuming that during the three‐night campaign all individuals inhabiting the area sampled are captured at least once, small mammal relative abundance was estimated as the number of different individuals captured throughout the whole campaign, an index that has been proved to give accurate population estimates (Torre et al., 2003).

### 2.4 Data analysis

Data analysis is divided in two main sections: (a) species daily activity patterns (mesocarnivores and small mammals) and (b) predator RAI as a function of prey abundance. In both sections, we also included the effect of season by dividing the information in two groups according to the astronomical season in which it had been obtained: spring–summer (before 23 September 2018 and after 20 March 2019) or autumn–winter (between 24 September 2018 and 19 March 2019).

#### 2.4.1 Daily activity patterns

Data were pooled from independent detections (contacts) of all camera trapping sites to calculate daily activity patterns of mesocarnivore species and small mammals. Firstly, detection times were extracted and transformed into radians (2*π* radians = 24 hr) before fitting a Kernel density function to them for each species and season with R package *overlap* (Ridout & Linkie, 2009). By comparing Kernel density functions in pairs, activity overlap indices were calculated in three different contexts: (a) overlap between seasons within a species, (b) overlap of mesocarnivore species within a season, and (c) overlap of a mesocarnivore species and small mammals within a season. Activity overlap indices correspond to the area shared by the two functions compared, thus normally taking values from zero to one: the lower the value, the more different activity patterns are. Here, indices were calculated by means of Dhat 4 and Dhat 1 equations according to the sample sizes obtained: for pairwise comparisons in which the smallest sample size was lower than 50 (a total of 16 comparisons out of 36), Dhat 1 was used as recommended by Meredith and Ridout (2018), whereas Dhat 4 was used in the other comparisons. When using Dhat 4 index, we took 1.0 as bandwidth, and for Dhat 1, 0.8 was applied (Ridout & Linkie, 2009). Bandwidth is the parameter that regulates the adjustment of the density functions to the times observed; resulting in spikier or softer curves (Rovero & Zimmermann, 2016). Finally, for Dhat 1 calculations, density was estimated in 144 equally spaced points (one every 10 min), a sufficient amount to obtain accurate estimates (Ridout & Linkie, 2009). Confidence intervals (95%) for overlap indices were estimated by bootstrapping 1,000 samples from the Kernel functions and calculating the overlap index for each iteration within each pairwise comparison by using the same R package *overlap* (Ridout & Linkie, 2009). Smooth bootstraps (i.e., values are taken from the density function instead of from the observed times) were used as they take into account the probability of animals being active and not detected (Rovero & Zimmermann, 2016). Due to excessively low seasonal mesocarnivore sample sizes obtained in several camera trapping sites (spring–summer: *n* = 30.94 ± 19.61, autumn–winter: *n* = 21.89 ± 18.08; mean detections site^−1^ ± *SD*), it was not possible to calculate and compare patterns among different sites, hence activity overlap was only assessed at a large scale.

To test whether two activity patterns could be considered as significantly different, we used R package *activity* (Rowcliffe, 2019). With this purpose, a null distribution of 1,000 random overlap indices was created by using bootstrap samples that contained values taken indiscriminately from any of the two density distributions to be contrasted. Then, the observed or “true” overlap index was compared to this null distribution to check the probability that it had arisen by chance (*p*). We considered that activity patterns were significantly different when *p* < .05.

#### 2.4.2 RAI and prey availability

RAI per camera trapping site was calculated for each mesocarnivore species as the number of independent detections per 100 days of camera trapping operation (Davis, Kelly, & Stauffer, 2011), assuming species are equally detectable across time. RAIs were calculated for the whole year and for every season, as well as for each mesocarnivore species and for all of them together. Before fitting the models, species year‐round RAIs per site were tested for spatial autocorrelation by means of Moran's *I* index—using mean coordinates obtained from the different camera positions within each site to calculate distance between camera trapping sites—and resulted in no significant effects (see Appendix S1). Besides, to test for differences in detection rates between both camera trap models (i.e., Browning and Cuddeback), we did a permutation test for the mean differences in mesocarnivore year‐round RAI per site. Specifically, we generated a null distribution of random differences obtained by permuting camera models among sites for 999 times. We could then compare the observed mean difference between models to the ones expected by chance and calculate *p* as the number of samples from the null model that were greater in absolute value than the observed difference. Overall, the effect of camera model on detection rate was not significant (Appendix S2). Finally, we checked that human frequentation was relatively low in most of the sites (Appendix S3) and linear mixed models (LMM) indicated that human pressure did not affect mesocarnivore seasonal activity (Appendix S4).

Small mammal availability effect on mesocarnivore activity was tested by means of linear mixed models (LMM). For each mesocarnivore species, its seasonal RAI per site was taken as the response variable, while season and small mammal relative abundance—gathering all the species in a single variable—were the main fixed explanatory variables. Besides, the RAI of the other four mesocarnivore species was also included as a fixed covariate to control for differences in detection rates caused by camera position. Finally, camera trapping site was added as a random factor. Before modeling, as continuous variables had been measured in different units, they were scaled by calculating the number of standard deviations at which each value was located from the variable mean. For each species, we constructed models from all possible combinations of explanatory variables by using the *dredge* and *lmer* functions from R packages *MuMIn* (Bartoń, 2015) and *lme4* (Bates, Mächler, Bolker, & Walker, 2015), respectively. Then, we calculated cumulative AICc weights of the variables (*importance* function from *MuMIn* R package) in order to determine which of them were the most likely to influence mesocarnivore RAI: the ones with a cumulative AICc weight ≥ 0.50 (Barbieri & Berger, 2004). We used the correction of Akaike's Information Criterion (AICc), as it is more suitable for small sample sizes. Finally, species most parameterized models (saturated) were checked for normality and homoscedasticity of the residuals. In this analysis, only camera trapping sites located at less than 3 km from a small mammal sampling plot were used (*n* = 13, Appendix S5), a distance easily covered by mesocarnivores according to their home ranges (Ferreras et al., 2016; Monterroso et al., 2009; Rosalino et al., 2004; Santos‐Reis et al., 2004). Besides, if a site had more than one small mammal sampling plot within this 3 km buffer (*n* = 4), small mammal abundance was calculated as the mean of these plots. Generally, habitat features of the location where the small mammal plot was set were similar to the ones characterizing the camera trapping sites associated with it.

## 3 RESULTS

**Figure 3:**
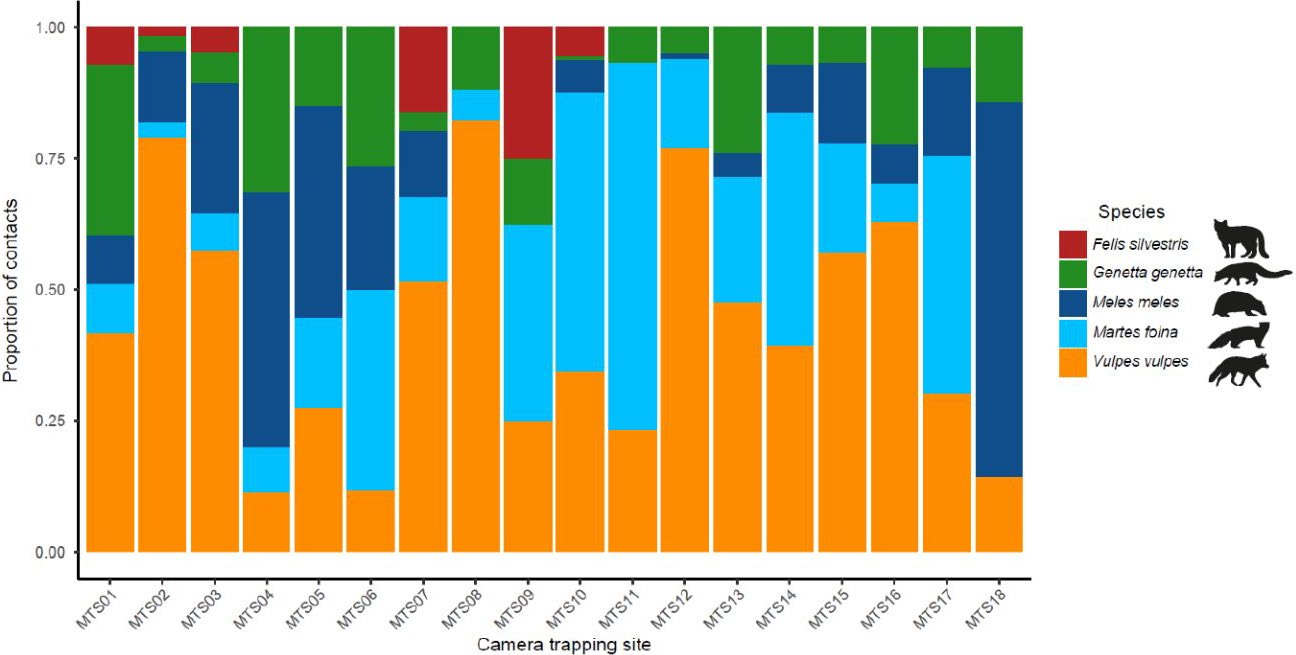
Proportion of independent camera trapping detections (contacts) for each mesocarnivore species in each camera trapping site

During the whole camera trapping period, 951 independent mesocarnivore detections were obtained (*n* = 52.83 ± 29.47; mean detections site^−1^ ± *SD*). Each species appeared in almost all the 18 camera trapping sites, except for the wildcat (Figure 3): red fox (18 sites: *n* = 456, 47.95% of mesocarnivore detections), stone marten (17 sites: *n* = 231, 24.29%), European badger (15 sites: *n* = 134, 14.09%), common genet (18 sites: *n* = 103, 10.83%), and European wildcat (6 sites: *n* = 27, 2.84%). In spring–summer, cameras captured 58.57% (*n* = 557) of the total mesocarnivore detections, for a 41.43% (*n* = 394) in autumn–winter. Camera trapping detection of small mammals followed the opposite pattern, with 34.65% (*n* = 220) of independent detections associated to spring–summer and 65.35% (*n* = 415) to autumn–winter. However, their detections were concentrated in only 9 of the 18 sites overall. Beyond the mentioned taxa, six other wild mammal species were captured during the sampling (Appendix S6).

### 3.1 Daily activity patterns

**Figure 4:**
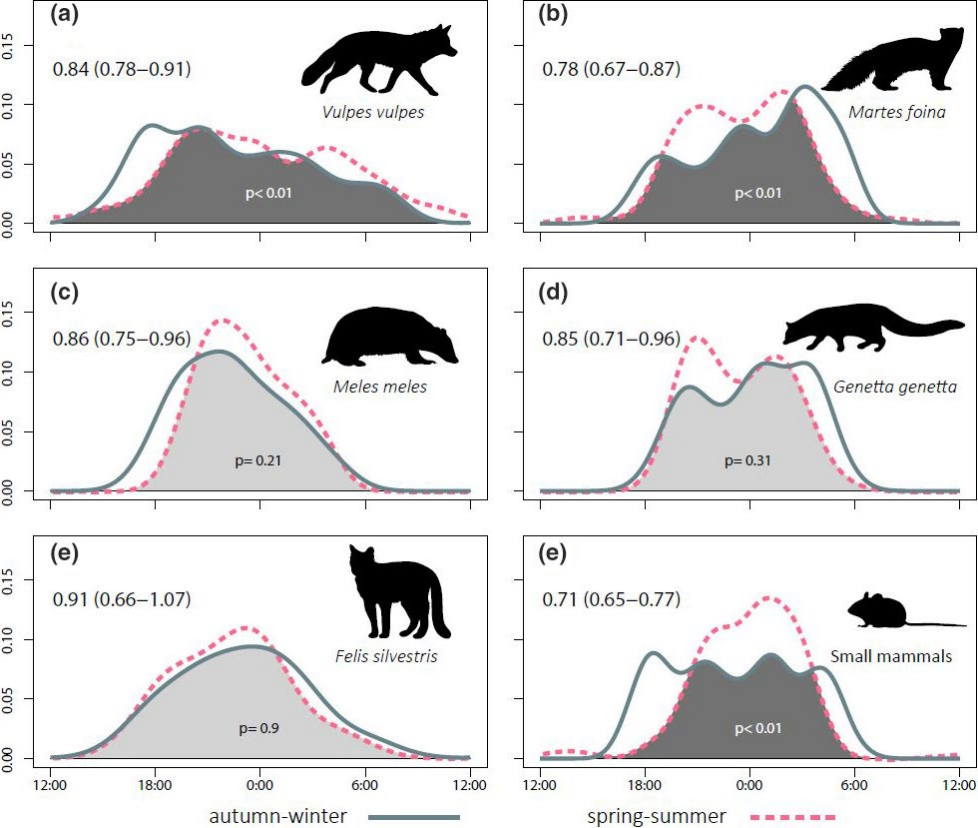
Daily activity patterns of the five mesocarnivore species (a–e) and small mammals (f). Line shape and colour according to the season (legend at the bottom). At the top left corner of each panel, Dhat 4 (a, b, c, f) or Dhat 1 (d, e) activity overlap indices obtained by comparing the pattern of both periods, with its 95% confidence interval. At the bottom, *p* associated with the overlap index, testing for significant differences between seasonal activity patterns. Hours are expressed in UTC and midnight is the central hour

As expected, the five mesocarnivore species and the small mammals exhibited a clear nocturnal activity pattern (Figure 4). During the whole year, red fox was the species with the largest activity level—*that is*, its activity pattern spanned more hours than the rest. Stone marten and genet had an irregular activity pattern, with two clear peaks in spring–summer. Finally, badger and wildcat showed a unimodal pattern with the activity peak centered between 21:00 hr and midnight solar time (Figure 4).

Regarding daily activity pattern variations, red fox and stone marten were the only mesocarnivore species that showed significant differences between seasons. However, these shifts did not occur in the same direction: while red fox began and reduced its activity earlier during autumn–winter, stone marten increased its activity around dawn the same season (Figure 4a,b). Despite not showing significant differences, the daily activity of the badger seemed to increase toward dusk in autumn–winter (Figure 4c), a similar process as the red fox, whereas genet daily activity shift tended to imitate the stone marten one (Figure 4d). Finally, the wildcat was the mesocarnivore with least activity pattern differences between seasons (Figure 4e). On the other hand, the small mammal population showed a considerably larger activity level in autumn–winter—around three more hours—than in spring–summer. During the latter season, their activity showed an only and important peak at 02:00 hr solar time (Figure 4f).

**Figure 5:**
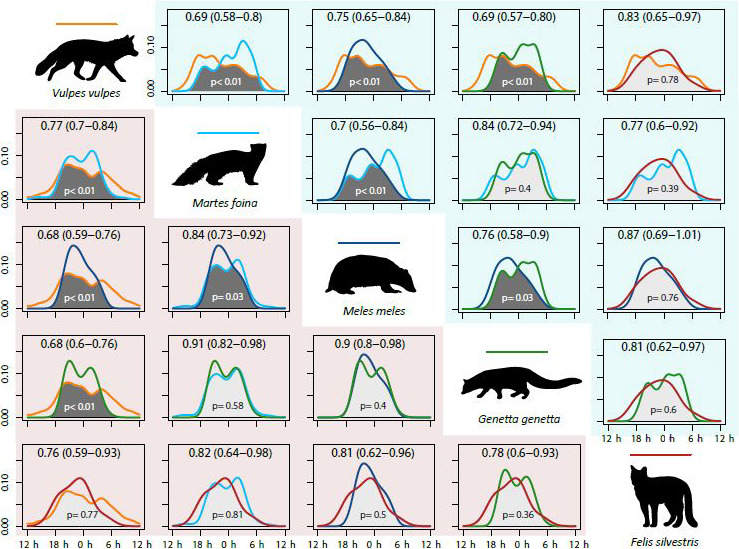
Comparisons between the daily activity patterns of target mesocarnivore species in spring–summer and autumn–winter, resulting in 10 comparisons per season. Graphs on red background (left side) belong to spring–summer, whereas graphs on blue (right side) belong to autumn–winter; function colors in accordance with species (line colour on each draw). At the top of each graph: Dhat 4 (or Dhat 1 for wildcat: both seasons; and genet: autumn–winter) activity overlap indices obtained with their 95% confidence interval. At the bottom: *p* associated with the overlap index, testing for significant differences. Hours are expressed in UTC and midnight is the central hour

When comparing mesocarnivore species to one another, red fox was the species that temporally overlapped the least with the other four mesocarnivores in both seasons (Figure 5). Overlap indices also showed that stone marten, badger, and genet temporally overlapped more among them in spring–summer than in autumn–winter, whereas red fox and wildcat comparison followed the opposite pattern. All comparisons between red fox and other mesocarnivore species, except for the wildcat, resulted in daily activity patterns being significantly different (Figure 5). On the other side, stone marten and genet were the species with the most similar daily routines on average (Figure 5).

**Figure 6:**
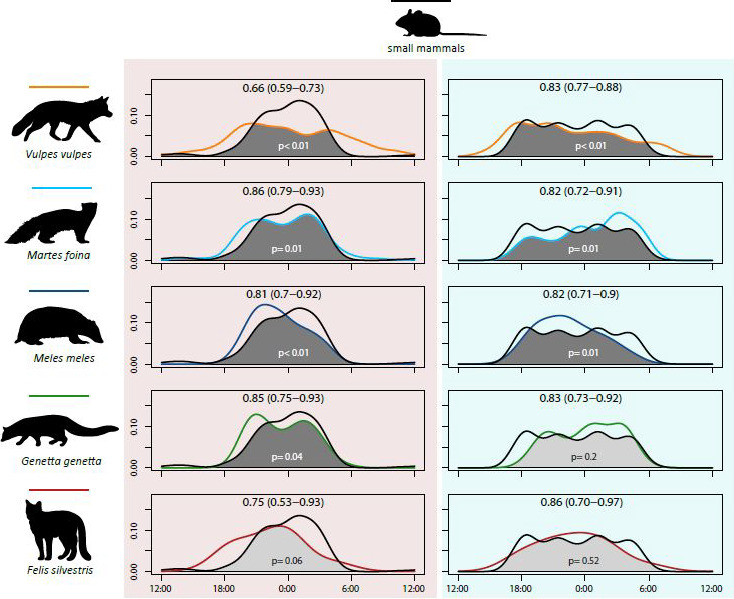
Comparisons between the daily activity patterns of target mesocarnivore species and small mammals in spring–summer and in autumn–winter, resulting in five comparisons per season. Graphs on red background (left) belong to spring‐summer, whereas graphs on blue (right) belong to autumn–winter; function colors in accordance with species (line colour on each draw). At the top of each graph: Dhat 4 (or Dhat 1 for wildcat: both seasons; and genet: autumn‐winter) activity overlap indices obtained with their 95% confidence interval. At the bottom: *p* associated with the overlap index, testing for significant differences. Hours are expressed in UTC and midnight is the central hour

Activity overlap indices resulting from the comparisons between mesocarnivore and small mammal patterns showed relevant differences (Figure 6). Stone marten and genet were the species that, in spring–summer, overlapped the most with this prey. On the other hand, overlap values became more homogenous among the different predator species in autumn–winter.

### 3.2 RAI and prey availability

**Figure 7:**
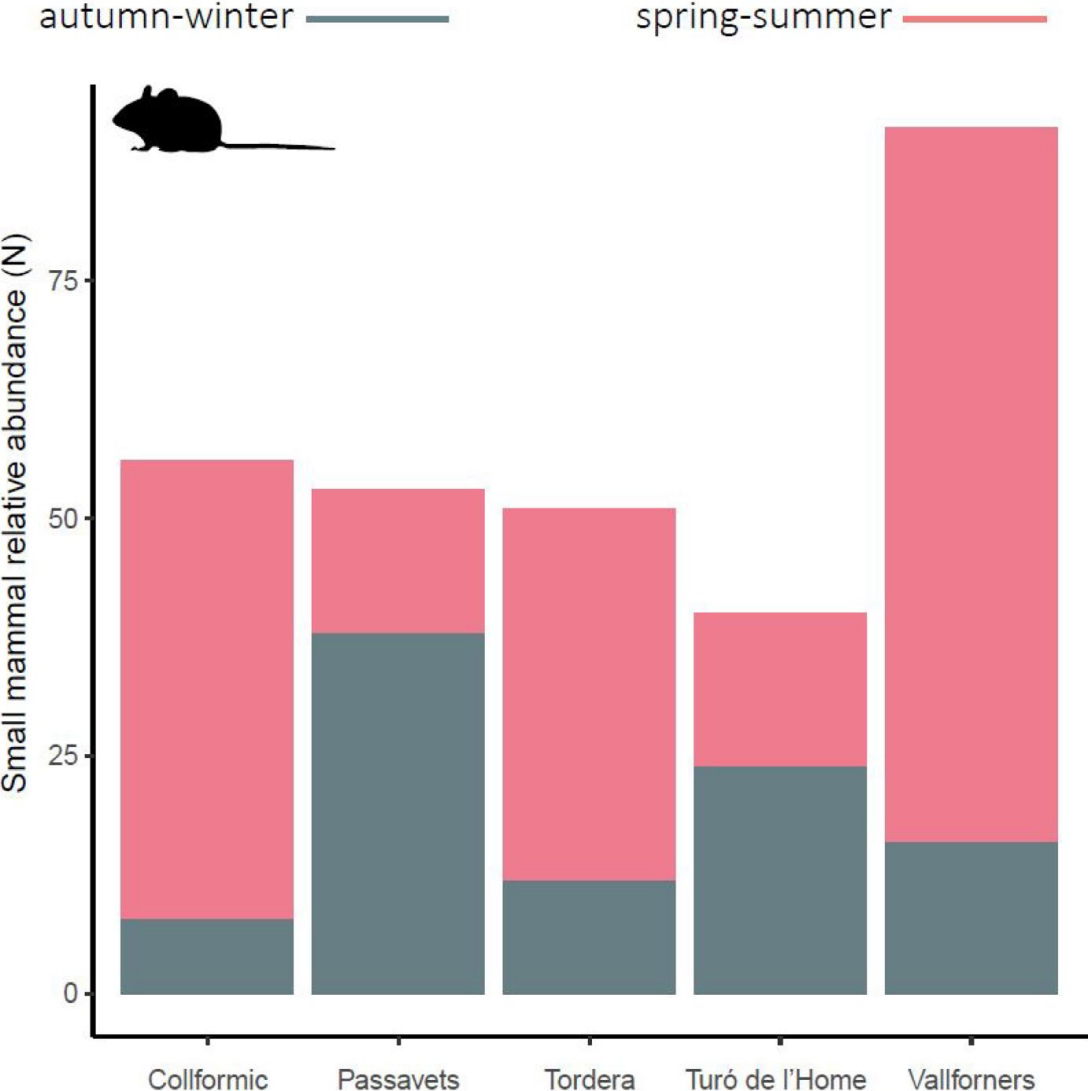
Small mammal relative abundance per survey plot. Bars coloured according to the season (legend at the top)

Gathering the five small mammal sampling plots, 291 individuals of 7 species were captured (see Data Accessibility Statement). The number of captures varied more between seasons than among different sampling plots (Figure 7). However, two distinct population dynamics were seen: The two plots located above 1,400 m (“Passavets” and “Turó de l’Home”) showed larger small mammal abundance in autumn–winter, whereas the other plots (550–1,200 m) were more productive in spring–summer.

**Figure 8:**
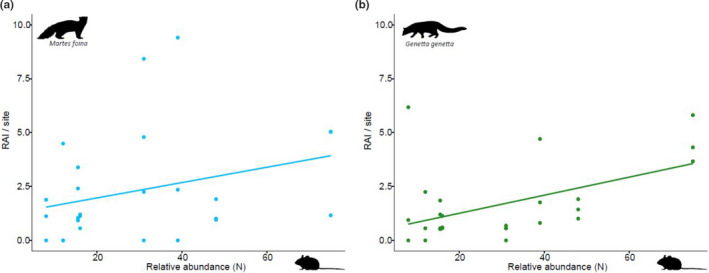
Effect caused by small mammal relative abundance on two mesocarnivore RAI models—stone marten (a) and genet (b). On the *y*‐axis: stone marten or genet RAI per site, calculated as the number of independent detections obtained in 100 operational days. On the *x*‐axis: small mammal relative abundance per plot, calculated as the number of different individuals captured

**Figure 9:**
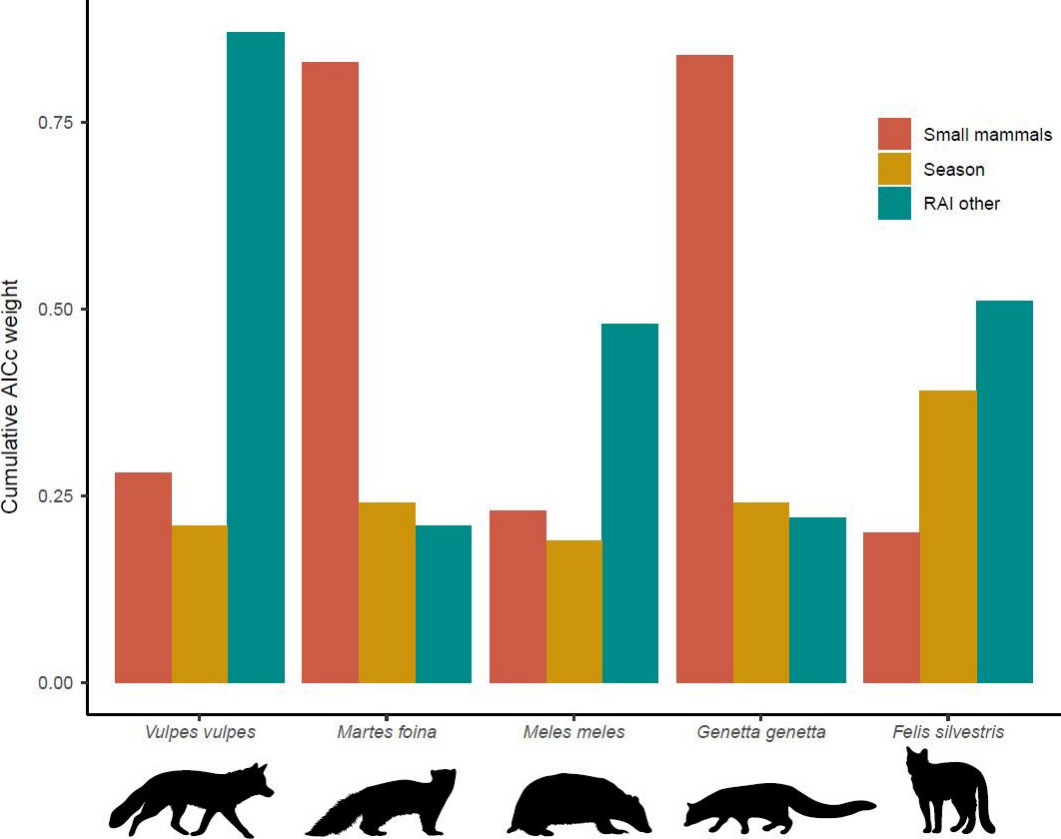
Cumulative AICc weight for the explanatory variables that were considered as candidates to influence seasonal RAI per site of mesocarnivores and were thus used to construct models with all combinations of variables for each species. *RAI other: RAI obtained by gathering the other four carnivore species

In mesocarnivore seasonal RAI models, stone marten and genet activity was positively influenced by prey availability (Figures 8 and 9), whereas the rest of predator species were not affected by it (Figure 9). Other mesocarnivore RAI was a relevant explanatory variable for the red fox, indicating that the canine showed more activity at sites where its competitors were frequently detected (Table 1, Figure 9). To a lesser degree, this variable also influenced positively badger and wildcat RAI (Table 1, Figure 9). Finally, season did not appear to have any meaningful role according to its cumulative AICc weights (<0.40) (Table 1).

**Table 1:** Model β coefficients (*SE* in brackets) of the most parsimonious model (lowest AICc) that included each variable and their respective cumulative AICc weights (in bold, AICc w+ > 0.50)

Species	Model parameters	Intercept	Small mammals	Season	RAI other
*Vulpes vulpes*	Coefficients (β)	1.36 (0.40)	−0.28 (0.26)	0.13 (0.44)	1.35 (0.48)
	AICc w+	–	0.28	0.21	**0.87**
*Martes foina*	Coefficients (β)	−0.09 (0.14)	0.21 (0.07)	−0.15 (0.17)	0.06 (0.09)
	AICc w+	–	**0.83**	0.24	0.21
*Meles meles*	Coefficients (β)	0.03 (0.16)	0.12 (0.14)	0.01 (0.27)	0.27 (0.15)
	AICc w+	–	0.23	0.19	0.48
*Genetta genetta*	Coefficients (β)	−0.24 (0.07)	0.21 (0.07)	0.06 (0.17)	0.05 (0.07)
	AICc w+	–	**0.84**	0.24	0.22
*Felis silvestris*	Coefficients (β)	−0.52 (0.06)	−0.01 (0.06)	−0.18 (0.11)	0.09 (0.05)
	AICc w+	–	0.20	0.39	**0.51**

Note

Linear mixed models (LMM) were used, with mesocarnivore species seasonal RAI per site as response variable. Autumn–winter season is taken as the reference level for the factor Season. *RAI other: RAI obtained by gathering the other four carnivore species. *SE*: standard error. *AICc*: corrected Akaike's information criterion. *w+*: cumulative weight.

## 4 DISCUSSION

Our study found that four out of five target mesocarnivore species — all except the wildcat — are widely spread across Montseny Natural Park. This broad distribution was expected according to the little preference shown by these species when selecting habitat features within Mediterranean forests (Barrull et al., 2013; Ferreras et al., 2016) and to the nonrandom locations of camera trapping sites. This spatial overlap observed at a large scale highlights the need to explore potential mechanisms that mesocarnivores use in order to reduce intraguild negative interactions. Although different degrees of diet specialization have been suggested as an important way to avoid competition of sympatric carnivores (Barrientos & Virgós, 2006), our results provide more information about the way some species use time and support the idea that temporal segregation could be an additional mechanism that favors the coexistence within complex Mediterranean carnivore guilds (see Monterroso et al., 2014). In this study, we have identified different activity pattern shapes, with the resulting variability in the levels of overlap among species. In addition, we found that some activity features at both daily and yearly levels are related to season and prey. Overall, time partitioning within a carnivore community should be considered when applying measures that aim to protect the guild as a whole (Davis et al., 2011; Lucherini et al., 2009).

The daily activity pattern of all target mesocarnivores is markedly nocturnal. However, some activity pattern shifts occur throughout seasons, as found in other studies (Barrull et al., 2013; Camps, 2008; Torretta et al., 2016), leading to seasonal variations in the degree of overlap among species. These shifts fall mainly on activity peaks, such as the case of the red fox—its activity peak moves toward dusk in autumn–winter, or on activity levels, such as the stone marten—it increases the amount of hours of activity during this same season. One of the most apparent results is that stone marten, badger, and genet temporal overlaps decrease in autumn–winter, a period when some carnivore feeding opportunities become more challenging (Carvalho & Gomes, 2004; Padial et al., 2002) and the increase of darkness hours enables more distinct daily activity patterns. On the other hand, activity of small mammals appears to be even more affected by season than mesocarnivore one. Being mostly nocturnal, they would follow the yearly variation of darkness hours. However, seasonal differences observed in this work should be regarded with caution, as more years of data would be required to test for the consistency of the patterns we report (see Marinho et al., 2020 as an example).

In general, activity overlap indices among mesocarnivore species obtained here are higher than in other carnivore community studies which performed a similar analysis (Bu et al., 2016; Curveira‐Santos et al., 2017; Karanth et al., 2017; Mukherjee et al., 2019): previous works where a few target species were essentially diurnal, allowing a broader temporal niche segregation within the guild. Here, despite carnivore activity is gathered at night hours, we still detected that each species differs in the way it temporally overlaps with the others. For instance, the red fox is active during a much larger amount of hours than other species do. This generalist behavior of the canine toward time use coincides with previous studies (Barrull et al., 2013; Ferreras et al., 2016) and could be associated with its largely flexible requirements in both spatial (O’Connell et al., 2006) and resource dimensions (Carvalho & Gomes, 2004; Ruiz‐Olmo & Aguilar, 1995). Nevertheless, as it is the most detected carnivore, intraspecific competition might be occurring and this could result in an apparent wider temporal niche at population level (Bolnick et al., 2003). To distinguish between these hypotheses, future work should try to identify individual specimens and study their activity patterns.

It is expected that species with similar sizes, habitats, and diets should have a low activity overlap to reduce negative interactions, such as the case of yellow‐throated marten (*Martes flavigula*) and masked palm civet (*Paguma larvata*) (Bu et al., 2016). In our case, despite these similarities exist between stone marten and genet (Barrientos & Virgós, 2006; Carvalho & Gomes, 2004), they appear to have the highest activity overlap, on average, of all mesocarnivore pairs analyzed. Consequently, results would suggest the presence of potential negative interactions, such as interference competition. In fact, Santos‐Reis et al. (2004) reported such situation, showing local territory exclusion between stone marten and genet individuals. Nevertheless, Barrientos and Virgós (2006) observed the existence of some discreet feeding mechanisms that could facilitate their coexistence, as the feeding behavior of the stone marten is more generalist than the one shown by the genet and they can sequentially use their shared resources. On the other hand, according to overlap indices observed, the wildcat seems to slightly avoid the activity peak of its closer competitor in terms of diet, the genet. However, this activity difference should be taken cautiously due to the low number of wildcat detections obtained, obeying its modest densities in the region (Sayol, Vilella, Bagaria, & Puig, 2018). Further analyses in the area should aim to check spatial and temporal overlap at once as we could not estimate local temporal segregation, which might be different from the one observed at a large scale (Monterroso et al., 2014). In addition, it could also be interesting to study other factors that can potentially have an influence on these carnivore activity patterns, such as land management or the presence of domestic animals (Ahumada et al., 2011; Curveira‐Santos et al., 2019; Rosalino, Macdonald, et al., 2005).

Activity pattern shapes in carnivore species not only might reflect interactions among them, but could also respond to prey behavior (Azevedo et al., 2018; Mukherjee et al., 2019). Therefore, as small mammals are a common prey for our target mesocarnivores (Carvalho & Gomes, 2004), we expected an association between mesocarnivore and small mammal daily activity patterns. In general, the activity overlap indices between predators and prey obtained here are relatively high in comparison with previous studies (Foster et al., 2013). This is especially the case of stone marten and genet in spring–summer and the wildcat in autumn–winter. Genet and wildcat predilection for small mammals (Ruiz‐Olmo & Aguilar, 1995; Torre et al., 2003) could partially explain their temporal association with this prey. In addition, predator–prey activity overlaps are more homogenous in autumn–winter than in spring–summer. In winter, consumption of small mammals tends to increase probably because other prey, namely birds and insects, as well as fruits, are usually less accessible (Carvalho & Gomes, 2004; Padial et al., 2002). Therefore, according to our results in the area sampled, autumn–winter could be the period when small mammals are targeted the most by mesocarnivores.

According to ecological theory of species competition, temporal and dietary niches should trade‐off with each other (Carothers & Jaksić, 1984). In our study area, for instance, one would expect that the activity overlap between badger and genet—the species with the most distinct diets (Rosalino, Loureiro, et al., 2005; Torre et al., 2003)—should be the highest, whereas the pairs genet/wildcat or red fox/stone marten should have a low overlap, according to their similar feeding requirements (Padial et al., 2002; Torre et al., 2003). However, these predictions were not met in our study at any of the seasons. On the other hand, theory suggests that the most specialized species at capturing small mammals (i.e., genet and wildcat) would temporally overlap significantly more with this prey than the other mesocarnivores. In this case, although prey overlap values of both species are relatively high, the genet was not the predator that overlapped the most with small mammals in any of the seasons and the wildcat only in autumn–winter, by a tight difference with respect to the other predators. Therefore, temporal and trophic niche dimensions seem to be contributing rather independently to the niche partitioning process of the studied guild.

Prey abundance is a factor that could explain variations in mesocarnivore RAIs. Here, we found that stone marten and genet RAIs were positively linked with small mammal abundance: They were also the predators with the highest temporal overlap with this prey in spring–summer. This positive effect was more expected for the genet than for the stone marten due to the important dependence on small mammals shown by the former (Torre et al., 2003). However, prey activity pattern coincidences with predators, as well as the effects of its availability, should be carefully regarded because other factors not analyzed here could be implicated in these predator–prey relationships, such as habitat features (Karanth et al., 2017).

We can conclude that there is a relatively high interspecific activity overlap among the Mediterranean mesocarnivores studied here, mainly due to their shared nocturnality. Nevertheless, some seasonal differences appear on species activity patterns, which can lead to either an increase or reduction of temporal segregation among them, potentially affecting intraguild coexistence. In this work, despite small mammal features seem to have an effect on stone marten and genet behavior, activity particularities shown by predators cannot be directly associated to their respective feeding habits. In general, in terms of time use, red fox appears as the most relaxed competitor of the guild studied, whereas stone marten and genet are the species with the highest activity overlap, eluding their similar size and habitat preferences. Cases like the latter should be carefully regarded and conservation efforts should guarantee enough optimal habitat for both predators (Gompper, Lesmeister, Ray, Malcolm, & Kays, 2016). Preserving entire carnivore guilds is essential to maintain the ecological stability of ecosystems, and here is where the establishment of large and diverse protected areas—such as Montseny Natural Park—together with the characterization of species requirements along different niche dimensions can be useful.

## CONFLICT OF INTEREST

The authors declare no competing interests.

## AUTHOR CONTRIBUTIONS

**Marc Vilella:** Conceptualization (equal); data curation (lead); formal analysis (lead); funding acquisition (supporting); methodology (lead); project administration (supporting); software (lead); writing–original draft (lead); writing–review and editing (equal). **Mariona Ferrandiz‐Rovira:** Conceptualization (equal); supervision (equal); validation (equal); writing–review and editing (equal). **Ferran Sayol:** Conceptualization (lead); data curation (supporting); formal analysis (supporting); funding acquisition (lead); methodology (lead); project administration (lead); supervision (equal); validation (equal); writing–review and editing (equal).

## Supporting information

Appendix S1‐S6

## Data Availability

The data generated for this study are available on Dryad (https://doi.org/10.5061/dryad.w6m905qn8).
