# Supplementary material for: Coexistence of predators in time: Effects of season and prey availability on species activity within a Mediterranean carnivore guild
Source: Ecol Evol. 2020 Sep 12;10(20):11408–22. doi: 10.1002/ece3.6778 (PMC7593183; doi:10.1002/ece3.6778)
Supplement: Supplementary file 1 — Appendix S1‐S6 [file ECE3-10-11408-s001.docx]

**APPENDIX 1**

**Spatial autocorrelation test (Moran’s *I*) on mesocarnivore species RAI**

| **Species** | **Moran’s I value (observed)** | **Moran’s I value (expected H0)** | **Moran’s I sd. (expected H0)** | ***P*** |
| --- | --- | --- | --- | --- |
| ***Vulpes vulpes*** | -0.02 | -0.06 | 0.08 | 0.66 |
| ***Martes foina*** | 0.00 | -0.06 | 0.06 | 0.32 |
| ***Meles meles*** | 0.04 | -0.06 | 0.08 | 0.19 |
| ***Genetta genetta*** | 0.04 | -0.06 | 0.08 | 0.22 |
| ***Felis silvestris*** | -0.03 | -0.06 | 0.06 | 0.64 |

**Note.** Mesocarnivore year-round RAIs per site were tested for spatial autocorrelation by using Moran’s *I* in order to validate the assumption of considering the 18 camera trapping sites as independent observations. Mean coordinates obtained from the different camera positions within each site (see *Materials and methods*) were used to calculate Euclidian distances between camera trapping sites.

**APPENDIX 2**

**Randomization test for differences in carnivore detection caused by using different camera models**


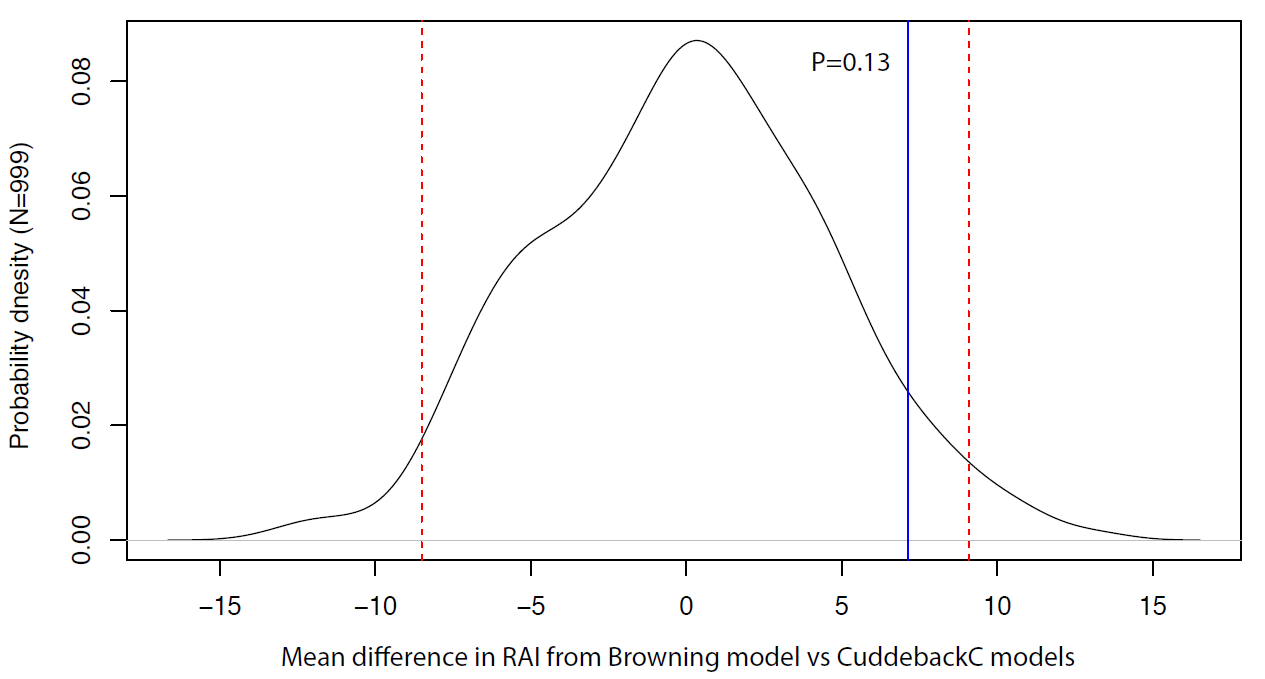


**Note.** We show the observed mean difference in mesocarnivore year-round RAI per site between the two camera models (blue line) and compare it to the expected distribution of mean differences generated by a null model of 999 permutations (red dashed lines show the 95% confidence interval of the null distribution). The test shows no significant difference in detection rate between both camera models (*P*=0.13).

**APPENDIX 3**

**Human frequentation at the different camera trapping sites**


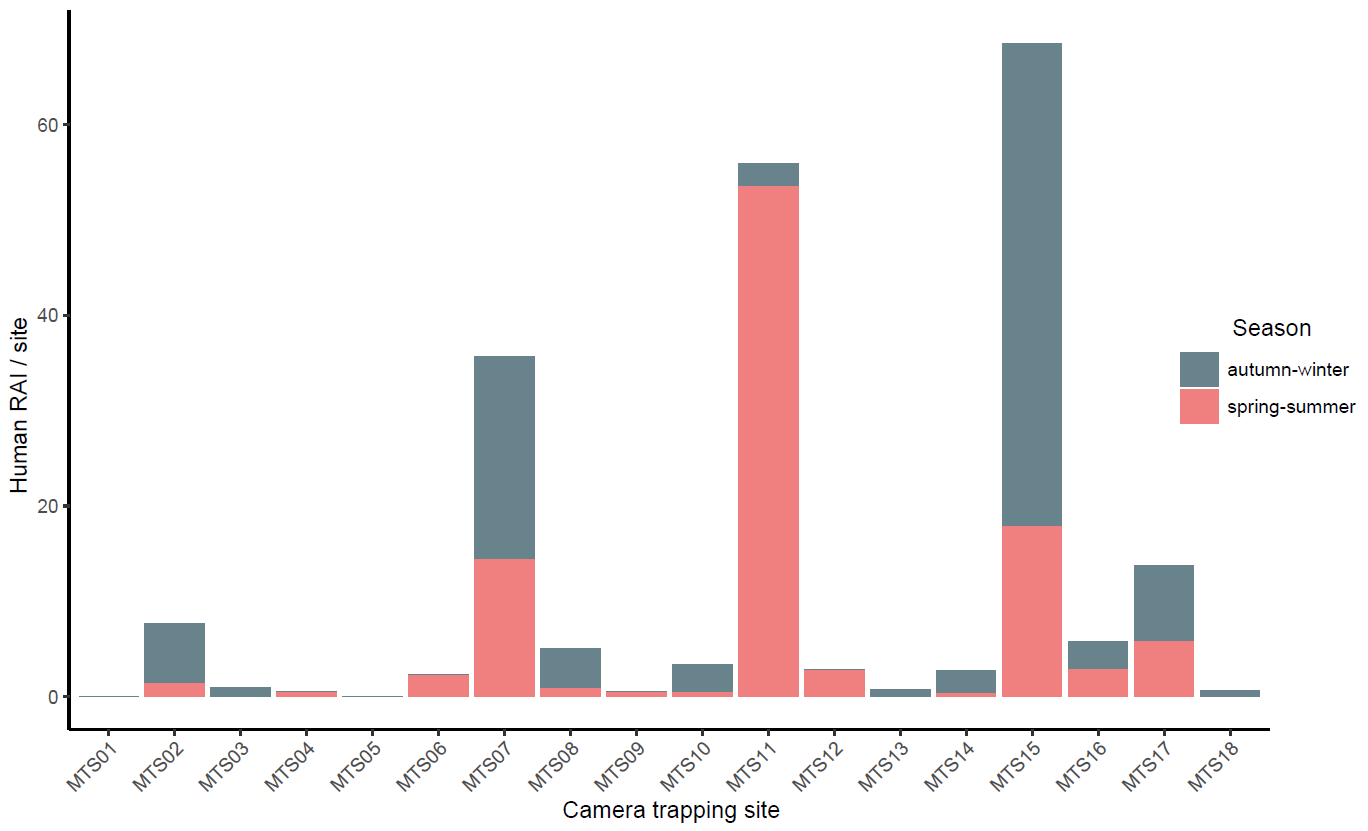


**Note.** Human seasonal RAI per site calculated from the contacts of people obtained by the cameras. Only three sites showed a relatively high human frequentation.

**APPENDIX 4**

**Linear mixed models (LMM) to test the effect of human frequentation on mesocarnivore detections**

| **Species** | **Intercept** | **Human RAI** | **AICc** | **∆AICc** |
| --- | --- | --- | --- | --- |
| *Vulpes vulpes* | **7.93** (1.47) | - | 257.22 | 0.00 |
|  | **7.66** (1.58) | 0.05 (0.11) | 259.59 | 2.37 |
| *Martes* *foina* | **3.78** (0.94) | - | 233.34 | 0.00 |
|  | **3.74** (1.04) | 0.01 (0.08) | 235.87 | 2.53 |
| *Meles meles* | **2.53** (0.59) | - | 188.46 | 0.00 |
|  | **2.60** (0.64) | -0.01 (0.04) | 190.93 | 2.47 |
| *Genetta* *genetta* | **1.71** (0.30) | - | 150.66 | 0.00 |
|  | **1.87** (0.33) | -0.03 (0.02) | 151.97 | 1.31 |
| *Felis silvestris* | **0.47** (0.20) | - | 119.78 | 0.00 |
|  | 0.42 (0.21) | 0.01 (0.02) | 122.05 | 2.27 |

**Note**. Model coefficients (SE in brackets) obtained from the linear mixed models (LMM) applied to each mesocarnivore species, with their seasonal RAI per site as response variable and human RAI as the only covariate. Models are ranked in ascending order for each species according to their AICc value. Values in bold indicate coefficients that are significantly different from zero (*P* < 0.05).

**APPENDIX 5**

**Selection of camera trapping sites to include in the RAI models**

**Note.** Euclidian distances (in meters) between each camera trapping site and its closest small mammal sampling plot. Mean coordinates obtained from the different camera positions within each site (see *Materials and methods*) were used to calculate these distances. Camera trapping sites located at more than 3 km from the closest small mammal plot were discarded (in grey), thus models included 13 of the 18 sites.

**APPENDIX 6**

| **Site** | ***Capreolus capreolus*** | ***Felis silvestris*** | ***Genetta genetta*** | ***Lepus europaeus*** | ***Martes foina*** | ***Meles meles*** | ***Small mammals*** | ***Mustela nivalis*** | ***Oryctolagus cuniculus*** | ***Sciurus vulgaris*** | ***Sus scrofa*** | ***Vulpes vulpes*** |
| --- | --- | --- | --- | --- | --- | --- | --- | --- | --- | --- | --- | --- |
| **MTS01** | 0 | 0.78 | 3.62 | 2.84 | 1.03 | 1.03 | 20.67 | 0 | 0 | 0.26 | 26.36 | 4.65 |
| **MTS02** | 0.3 | 0.3 | 0.61 | 0.61 | 0.61 | 2.74 | 0 | 0 | 0 | 0 | 15.55 | 16.16 |
| **MTS03** | 5.08 | 1.27 | 1.59 | 9.52 | 1.9 | 6.67 | 0 | 0 | 3.49 | 0 | 81.59 | 15.56 |
| **MTS04** | 0.29 | 0 | 3.14 | 0 | 0.86 | 4.86 | 0 | 0 | 0 | 0 | 8.29 | 1.14 |
| **MTS05** | 0.44 | 0 | 3.08 | 1.32 | 3.52 | 8.37 | 0 | 0.44 | 0 | 0 | 27.75 | 5.73 |
| **MTS06** | 1.04 | 0 | 2.34 | 1.56 | 3.39 | 2.08 | 0 | 0 | 0 | 0.26 | 23.18 | 1.04 |
| **MTS07** | 0 | 3.93 | 0.87 | 3.49 | 3.93 | 3.06 | 0 | 0 | 0 | 0 | 21.4 | 12.66 |
| **MTS08** | 9.92 | 0 | 0.79 | 5.56 | 0.4 | 0 | 0 | 0 | 0 | 0 | 63.89 | 5.56 |
| **MTS09** | 1.36 | 1.08 | 0.54 | 0 | 1.63 | 0 | 0 | 0 | 0 | 0.81 | 1.9 | 1.08 |
| **MTS10** | 7.89 | 1.69 | 0.28 | 1.97 | 16.9 | 1.97 | 18.03 | 0 | 0 | 1.97 | 48.45 | 10.99 |
| **MTS11** | 0.28 | 0 | 0.56 | 0 | 5.92 | 0 | 36.62 | 0 | 0 | 0 | 17.18 | 1.97 |
| **MTS12** | 4.79 | 0 | 1.13 | 0 | 3.94 | 0.28 | 23.66 | 0 | 0 | 5.63 | 62.25 | 18.03 |
| **MTS13** | 11.37 | 0 | 4.37 | 9.62 | 4.37 | 0.87 | 16.03 | 0 | 0 | 5.25 | 35.86 | 8.75 |
| **MTS14** | 6.43 | 0 | 0.77 | 0.26 | 4.88 | 1.03 | 50.64 | 0.77 | 0 | 6.68 | 25.19 | 4.37 |
| **MTS15** | 8.2 | 0 | 2.21 | 23.34 | 6.94 | 5.05 | 5.36 | 0 | 0 | 2.84 | 29.97 | 18.93 |
| **MTS16** | 0 | 0 | 3.45 | 0 | 1.15 | 1.15 | 2.01 | 0.29 | 0 | 0.29 | 8.33 | 9.77 |
| **MTS17** | 0.86 | 0 | 1.15 | 10.34 | 6.9 | 2.59 | 0.29 | 0.29 | 0 | 0 | 16.95 | 4.6 |
| **MTS18** | 0.37 | 0 | 0.37 | 0 | 0 | 1.85 | 0 | 0 | 0 | 0.37 | 2.96 | 0.37 |

**Wild mammal species detected in each camera trapping site during the study**

**Note**. Values correspond to year-round RAI per site; see *Materials and methods* for details. Small mammal species were not identified due to difficulties in telling apart some taxa from camera trapping images.
